# A virtual source model for Monte Carlo simulation of helical tomotherapy

**DOI:** 10.1120/jacmp.v16i1.4992

**Published:** 2015-01-08

**Authors:** Jiankui Yuan, Yi Rong, Quan Chen

**Affiliations:** ^1^ University Hospitals Case Medical Center Cleveland OH; ^2^ Department of Radiation Oncology The Ohio State University Wexner Medical Center Columbus OH; ^3^ Department of Radiation Oncology University of Virginia Charlottesville VA USA

**Keywords:** Monte Carlo simulation, radiation therapy, tomotherapy, virtual source modeling

## Abstract

The purpose of this study was to present a Monte Carlo (MC) simulation method based on a virtual source, jaw, and MLC model to calculate dose in patient for helical tomotherapy without the need of calculating phase‐space files (PSFs). Current studies on the tomotherapy MC simulation adopt a full MC model, which includes extensive modeling of radiation source, primary and secondary jaws, and multileaf collimator (MLC). In the full MC model, PSFs need to be created at different scoring planes to facilitate the patient dose calculations. In the present work, the virtual source model (VSM) we established was based on the gold standard beam data of a tomotherapy unit, which can be exported from the treatment planning station (TPS). The TPS‐generated sinograms were extracted from the archived patient XML (eXtensible Markup Language) files. The fluence map for the MC sampling was created by incorporating the percentage leaf open time (LOT) with leaf filter, jaw penumbra, and leaf latency contained from sinogram files. The VSM was validated for various geometry setups and clinical situations involving heterogeneous media and delivery quality assurance (DQA) cases. An agreement of <1% was obtained between the measured and simulated results for percent depth doses (PDDs) and open beam profiles for all three jaw settings in the VSM commissioning. The accuracy of the VSM leaf filter model was verified in comparing the measured and simulated results for a Picket Fence pattern. An agreement of <2% was achieved between the presented VSM and a published full MC model for heterogeneous phantoms. For complex clinical head and neck (HN) cases, the VSM‐based MC simulation of DQA plans agreed with the film measurement with 98% of planar dose pixels passing on the 2%/2 mm gamma criteria. For patient treatment plans, results showed comparable dose‐volume histograms (DVHs) for planning target volumes (PTVs) and organs at risk (OARs). Deviations observed in this study were consistent with literature. The VSM‐based MC simulation approach can be feasibly built from the gold standard beam model of a tomotherapy unit. The accuracy of the VSM was validated against measurements in homogeneous media, as well as published full MC model in heterogeneous media.

PACS numbers: 87.53.‐j, 87.55.K‐

## I. INTRODUCTION

Helical tomotherapy^(1)^ delivers highly modulated radiation intensity with binary multileaf collimators (MLCs) and narrow fan beams during continuous gantry rotation with simultaneous couch translation. It is a special form of intensity‐modulated radiation therapy (IMRT) which involves complex delivery pattern and irregular beam apertures shown as a sinogram. It has been demonstrated that treatment plans with excessive small irregular beams may lead to significant dose calculation errors in inhomogeneous regions using correction‐based algorithms or convolution–superposition algorithms.^2^, ^3^, ^4^, ^5^, ^6^, ^7^, ^8^, ^9^, ^10^, ^11^, ^12^, ^13^, ^14^, ^15^ Monte Carlo (MC) simulation is still considered the gold standard method for accurate dose calculations.

Previously reported MC models for tomotherapy directly simulate physical interactions of particles within the linac head, using the precise information of each component including location, size, shape, and material components.^16^, ^17^, ^18^, ^19^, ^20^ Traditional MC codes, such as MCNP4C,^(21)^ PENELOPE,^(22)^ EGSnrc, BEAMnrc, and DOSXYZnrc,^23^, ^24^ created phase‐space files (PSFs) by simulating all interactions in the linac head with a considerable amount of particles (on the order of $10^{8}$ or more) at the exit phase of the linac.

The PSFs created by full MC simulation contain the most accurate physical description of the radiation beam exited from the linac, provided that the information characterizing every component in the linac head is precise in terms of detailed geometric and material specifications. In reality, the detailed mechanic drawings of a linac are most likely confidential and secured by the manufacturer. Of the four MC models of tomotherapy in literature, only Jeraj et al.^(16)^ and Sterpin et al.^(17)^ gained access to the proprietary information from their close connection with the vendor. However, to our best knowledge, their MC models are not shared in the public domain, thus prohibiting others from directly adopting their models for subsequent research. Belec et al.^(20)^ built their model based on direct caliper measurements of two tomotherapy units, which may be subject to measurement uncertainties. In addition, some of the input information required in the MC simulations, such as the incident electron energy spectrum and the focal spot size, may not be precisely modeled, even for those who have access to the proprietary information. This led to possible modeling uncertainties in photon spectrum and focal spot size, which explained the differences in the results reported by the Jeraj and Sterpin studies. The correction of focal spot size from their studies was estimated from 1.4 mm^(17)^ to 1.1 mm,^(25)^ which was still different from the direct measurement.^(26)^

In addition to the lack of detailed specifications for linac components, TG‐105 outlines a few other limitations associated with the use of PSFs.^(27)^ The existence of latent variance^(12)^ dictates that the PSF size for obtaining acceptable uncertainty (1%–2% for a voxel size of $0.5\times 0.5\times 0.5\;{\text{cm}}^{3}$) is on the order of gigabytes (GB).^8^, ^27^, ^28^ Not only does the size of the PSF create storage and deployment issues, the slow speed of reading the PSF from a hard disk also creates a performance bottleneck.^(27)^

An alternative method for beam modeling is the virtual source model (VSM) approach.^(29)^ This method assumes particles emitted from the linac are originated from a single or multiple virtual sources with different geometries (e.g., point, disc, annulus). Fluence distribution and energy spectrum for each subsource can be reconstructed either from well‐commissioned PSFs or sets of measurements.^4^, ^6^, ^30^ The advantage of this approach is that it does not carry large‐size PSFs and the latent variance can be significantly reduced.^(31)^ Additionally, it is possible to derive the model from measurements alone without knowing the details of a linac design. Similar methods have been used for beam modeling in conventional dose calculation algorithms.^(26)^ However, there has not been any study applying the VSM to tomotherapy units. The purpose of this work is to demonstrate that our proposed tomotherapy VSM can be accurately employed in MC simulations for patient dose calculations.

## II. MATERIALS AND METHODS

### A. Tomotherapy VSM

**Figure 1:**
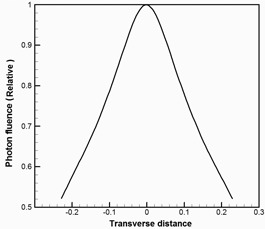
1D transverse fluence profile in tomotherapy VSM. Note that the transverse distance is not physical distance, but tangent of the off‐axis angle.

**Figure 2:**
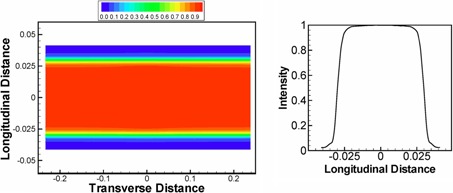
Illustration of 2D jaw profile used in tomotherapy VSM: (left) 2D jaw profile for 5.0 cm jaw width; (right) the longitudinal profile at the beam center (transverse distance=0). Note that the distances labeled in each axis are not physical distances, but tangents of the off‐axis angle.

Tomotherapy uses convolution–superposition (CS) techniques^32^, ^33^, ^34^ for intermittent dose calculations during optimization and final dose calculations. The gold standard beam model is stored in the XML files and associated binary files in the TPS machine archive. We hypothesized that the generalized three‐component source model for a conventional linac, which includes primary, first (for collimator), and second (for head) scatter source, can be simplified to a single‐source model for a tomotherapy linac due to its unique source design of eliminating the flattening filter^(29)^ and using thick primary collimator (23 cm thick tungsten).^(35)^ The peripheral dose, which is a measure of the scatter contribution, is an order of magnitude lower for tomotherapy than conventional linacs.^(35)^ In building our VSM model, the scattered photons from the linac head and beam limiting devices such as jaws and MLCs were measured together with the primary photon. The approximation used in our VSM model was that scattered photons were assumed to originate from the primary photon source. Electron contamination was excluded from calculation due to its negligible effect shown in the full MC model of tomotherapy.^(17)^ To account for the difference in penumbra and fluence from different jaw settings (1 cm, 2.5 cm, and 5 cm jaw size), profiles for each individual jaw size were kept as part of the VSM. The VSM included a 1D transverse profile C(x) (i.e., cone profile) and a 2D longitudinal profile $J_{\text{js}}(x,y)$ (i.e., jaw profile or jaw penumbra) for each jaw size. The x,y variables were defined as positive x pointing to patient's left and positive y pointing to the gantry. Both C(x) and $J_{\text{js}}(x,y)$ were normalized to 1 at the center of the beam axis and carried no units. Figure 1 demonstrates a typical transverse fluence profile, characterized as a cone shape due to lack of a flattening filter in the tomotherapy unit. Figure 2 shows the longitudinal profile of a 5 cm jaw size over the entire field. The x and y variables in C(x) and $J_{\text{js}}(x,y)$ are not physical distances, but tangents of the off‐axis angle. Using tangent values on the axes makes fluence profiles applicable to planes at any distance from the source. The 1D longitudinal profile at the beam center (x=0) is also plotted. Taking the jaw penumbra into account, the fluence map for the open fields (40 cm×1 cm, 2.5 cm, and 5 cm) can be reconstructed as

(1) $$f_{\text{js}}(x,y)=C(x)*J_{\text{js}}(x,y)$$

**Figure 3:**
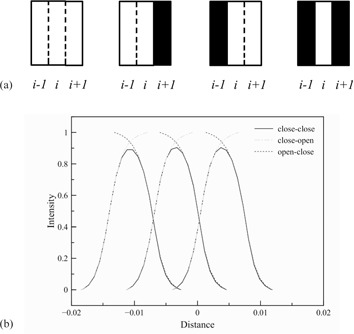
Four possible combinations (a) of the state of adjacent leaves (Leaf i−1 and i+1) of Leaf i, which are open‐open, open‐close, close‐open, and close‐close. Solid color illustrates the closed leaf. Example (b) of the leaf filter for three adjacent leaves.

In tomotherapy dose calculation, the effect of MLC modulation has been modeled by a transfer function called the “leaf filter”.^(36)^ Due to the tongue‐and‐groove (T&G) and penumbral blur effect, the actual fluence transmitted to a point under the direct path of a leaf of interest (LOI) is dependent on the state of its adjacent leaves. In earlier generation of tomotherapy machines, the effect of neighbor leaves was modeled with a scalar leaf fluence output factor (LFOF), which was the increase in total fluence under an LOI when adjacent leaves were open. In other words, LFOF was a factor that represented the effect on the fluence of a LOI from the open/close state of its neighbors. The fluence profile under an LOI was assumed to be a rectangular function. In new generations of tomotherapy units (HA4.x.) with the TomoDirect feature, the actual fluence profile of each LOI when adjacent leaves are opened or closed is recorded as the leaf filter. It is determined that only the state of the two direct adjacent leaves (one on each side) could impact the fluence profile of an LOI. The leaf filter is a profile that represents the fluence distribution for a given open/closed leaf configuration, and therefore it takes the fluence at the leaf boundaries into consideration. For an open LOI, there are four possible combinations of the state of the two adjacent leaves, which are “open‐open”, “open‐close”, “close‐open”, and “close‐close”, as shown in Fig. 3(a). Since only the adjacent leaves affect the fluence profiles of an LOI, the “open‐open” profile should not be different from the LOI fluence profile when all leaves are open. Therefore, the “open‐open” profile is not stored, as it can be sampled directly from the cone profile. Each leaf has its own leaf filter. The VSM contains 64 leaf filters as each leaf filter represents one MLC leaf. The “open‐close”, “close‐open”, and “close‐close” profiles for each LOI are normalized with respect to the cone profile. The leaf filters were measured with the on‐board MVCT detector at the time of machine commissioning and remeasured whenever the MLC or linac source was replaced. Figure 3(b) plotted the leaf filters of three adjacent leaves, indicating the enhanced total fluence (dashed lines) when one adjacent leave is open, compared to the “close‐close” profile (solid lines).

With applying the leaf filter, the fluence profile of a leaf configuration $s=\{s_{1},\;s_{2},\ldots s_{i}..\}$ for a given projection can be described as:

(2) $$L_{F}(s,x)=\sum_{i=1}^{64}\left\{A_{1i}*f_{ooi}(x)+A_{2i}*f_{cci}(x)+A_{3i}*f_{coi}(x)+A_{4i}*f_{oci}(x)\right\}$$

where $s_{i}$ is the LOT for leaf i, $f_{\text{ooi}}(x),f_{\text{cci}}(x),f_{\text{coi}}(x)$, and $f_{\text{oci}}(x)$, are “open‐open”, “close‐close”, “close‐open” and “open‐close” filters for leaf i, respectively, and *x* is the transverse coordinate defined previously. The coefficients $A_{1i},A_{2i},A_{3i},A_{4i}$ represent the open time spent in the above four states, which are normalized to the duration time of a projection, and can be calculated as:

(3) $$\begin{array}{l} A_{1i}=\text{min}(s_{i-1},s_{i},s_{i+1},)\\ A_{2i}=s_{i}-\text{max}(s_{i-1},s_{i+1},)\\ if\;s_{i+1}>s_{i-1},\;A_{3i}=s_{i}-A_{1i}-A_{2i},A_{4i}=0\\ else\;A_{3i}=0,\;A_{4i}=s_{i}-A_{1i}-A_{2i},A_{4i} \end{array}$$

Taking the beam limiting devices (primary collimator, jaws and MLC) into account, for each projection, the 2D fluence map under MLC can be modeled as s

(4) $$f_{\text{js}}(s,x,y)=C(x)\times J_{\text{js}}(x,y)\times L_{F}(s,x)$$

**Figure 4:**
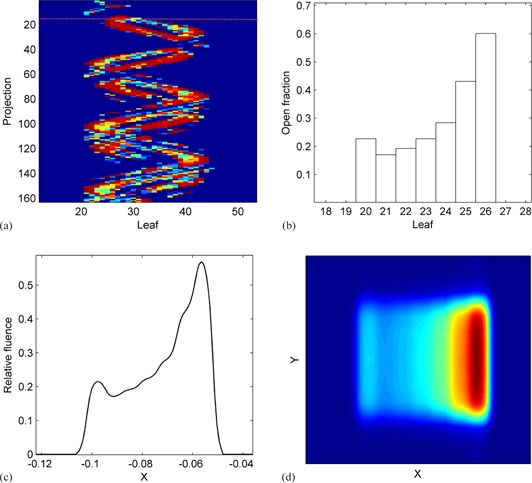
Example of a 2D fluence map created from the leaf sonogram: (a) leaf sonogram; (b) leaf open time as fractions of projection duration for the projection indicated with the red line in (a); (c) 1D fluence profile after applying the leaf filter to (b); (d) 2D fluence map after applying the cone profile and jaw penumbra to (c).

The equation means the fluence of a fan beam upon exiting the primary collimator is assumed to be a cone shape (C(x)) and independent of the Y direction. A fan beam is first collimated by the jaws, followed by further shaping with MLCs in a specific leaf pattern $\left(L_{F}(s,x)\right)$, which consists of open time for each individual leaf. The fluence at the jaw penumbra region is described by the jaw profile ($J_{\text{js}}(x,y)$) in both X and Y directions of the jaw. Therefore, $f_{\text{js}}(s,x,y)$ represents a 2D fluence map exiting the MLC, which can be generated accordingly given a leaf sinogram $s=\{s_{1},\;s_{2},\ldots s_{i}..\}$. Figure 4 illustrates the process of converting leaf sinogram to a 2D fluence map for one projection.

In MC simulations, random numbers are generated to sample the initial position and direction of the primary particles. The initial position of each particle is sampled from the fluence map $f_{\text{js}}(s,x,y)$ at the MLC exit plane. The origin of a particle is sampled from a double‐Gaussian distribution, reported in Chen et al.,^(25)^ at the target plane. It has been shown that the double‐Gaussian distribution provides the best agreement at the penumbra region.^(25)^ The direction of a particle is determined as the vector connecting the origin with the initial position. Note that in this scheme, particles that are scattered from collimators are assumed to originate from the target as well. However, as shown in our Results section below, this approximation did not produce significant errors, even in the penumbra region.

Another component that needs to be addressed in a VSM is the energy spectrum. Since tomotherapy uses a polyenergetic kernel for dose calculations, the energy spectrum is implicitly incorporated in the kernel. Therefore, the exported machine file does not provide the energy spectrum information. Jeraj et al.^(16)^ published the first energy spectrum for a tomotherapy unit and showed that the energy spectrum was comparable to Varian C linac spectra of the same nominal energy and had almost negligible off‐axis spectral dependence. The maximum energy was about 6 MeV and the average energy was around 1.49 MeV. The other published spectrum was from Sterpin's work,^(17)^ which was extracted from the PSF and showed clearly a peak at 511 keV due to electron–positron annihilation. The average energy was also around 1.4 MeV, with the maximum energy being 5.5 MeV. The apparent difference between those two published tomotherapy beam models highlights the challenges in modeling a linac even with the best knowledge of the linac design. In this work, we selected (arbitrarily) Jeraj's spectrum for our study and expressed it in parametric form with the function ${\text{aE}}^{0.5}+{\text{bE}}^{-0.5}+{\text{ce}}^{-E}+d$, where a=−0.352,b=−1.42,c=2.22, and d=1.437 for E (MeV)>0.25, for the flexibility of finetuning the spectrum by changing a few parameters. We found that this fitting curve resulted in a good agreement with measurements, while other sets of fitting parameters and equations can also be used.^(37)^

MC simulation outputs data in the unit of dose per simulated particle. In order to obtain absolute dose, a calibration factor needs to be determined. Specifically, the calibration factor M (particles/s), which converts the MC result to dose (Gy), is defined as $D_{m}=M\times T_{p}\times D_{\text{MC}}$, where $D_{m}$ (Gy) is the measured point dose, $T_{p}$ (s) is the total LOT (summed over every leaf and every projection), and $D_{\text{MC}}$ is the MC result (Gy/particle) at the same point.^(37)^ This factor can be determined by running the MC calculation for a simple plan and comparing the calculation result with measurements. Once the calibration factor M is determined, it can be used to convert the MC result to the dose distribution for all subsequent MC‐VSM calculations.

In summary, sinograms generated from the TPS were first exported through DICOM as part of the RTPLAN object. Leaf latency correction was applied to convert the programmed LOT to effective LOT.^(38)^ A fluence map was then created by incorporating the percentage open‐close leaf time with the leaf filter, jaw profile, and cone profile. The start position of a photon was sampled from the fluence map and its direction was the vector connecting its start position and a sampled position from the Gaussian distribution of the source. The photon energy was sampled from the spectrum function given above. TomoTherapy Hi ·Art 4.1 was used in our study.

### B. Geometry transformation

Since tomotherapy operates in a helical rotational fashion, geometry transform is needed to follow the particle in MC simulations. Our MC code adopts patient's DICOM coordinate system: the x‐axis is from patient's right to left, the y‐axis is from patient's anterior to posterior, and the z‐axis is from patient's inferior to superior. Tomotherapy, on the other hand, employs the IEC 61217 coordinate system, in which the $x_{\text{IEC}}$ axis points to patient's left, the $y_{\text{IEC}}$ axis points the gantry, and the $z_{\text{IEC}}$ axis points upwards. Different from conventional linacs, the couch angle and the collimator angle in tomotherapy are always zero. For a point $p_{b}=[x_{b},y_{b},{z_{b}]}^{T}$ in the coordinate system of the beam limiting device (MLC), the transformed point in patient's coordinate system can be expressed by

(5) $$T(x_{iso},y_{iso},z_{iso})*R_{xf}(90)*R_{z}(-\theta_{couch})*R_{y}(\beta_{gantry})*T(0,0,SAD)*R_{zf}(\theta_{col})*Pb$$

where $T(x_{\text{iso}},y_{\text{iso}},z_{\text{iso}})$ is an affine matrix that translates patient's coordinate system to couch coordinate system. $R_{x,y,z}\left(\beta \right)$ is the rotation matrix around the x, y, z axes, respectively, relative to the fix room coordinate system with angle β. At each projection, the central axis of the beam passes through an isocenter $\left(x_{\text{iso}},y_{\text{iso}},z_{\text{iso}}\right)$, where $\left(x_{\text{iso}},y_{\text{iso}}\right)$ are set at the CT image center, and $z_{\text{iso}}$ moves along the Z direction. The isocenter position and gantry angle of each projection can be obtained from the exported RTPLAN.

### C. Monte Carlo algorithm

The in‐house MC code for this work is a C++ implementation of DPM,^(39^) which is a fast MC program written in FORTRAN and designed specifically for radiation therapy. DPM is a well‐benchmarked MC code used in the medical physics community.^(39)^ Similar to DPM, Compton scattering, photoelectric ionization, and pair production were considered for photon transport in our MC code. Every interaction was modeled individually until the energy of the particle fell below a cutoff energy or the particle escaped the simulation volume. For electron transport, the class II condensed history method was used. Hard interactions, such as inelastic collisions and bremsstrahlung, were simulated explicitly for energies above certain cutoffs. The continuous slowing down approximation (CSDA) was employed for energy below the threshold, which was set to 200 keV for electrons. The cutoff energy for photons was set to 50 keV.^(39)^ Photon energies are deposited locally if they are lower than the cutoff energy. The code has been validated by comparing results for the ICCR heterogeneous slab phantoms^(40)^ with the published EGSnrc results as benchmarks, and for other phantom tests, as well. The Message Passing Interface (MPI) was implemented in the code, to take advantage of multiprocessor computing resource.

### D. Validation of the VSM

The validation of our VSM was performed by comparing the simulation (MC‐VSM) with the measurements that were done at the time of machine commissioning when the tomotherapy unit was installed. All measurements were taken with the Exradin A1SL ion chambers (Standard Imaging, Middleton, WI) in a water tank at 85 cm SSD. In order to minimize the volume averaging effect in large dose gradient regions during scanning, the A1SL ion chamber was used for its small inner diameter (4.05 mm) and active volume (0.056 cm^3^). For dose distributions, the EBT2 radiochromic films (Advanced Materials, Wayne, NJ) were used and scanned with a VIDAR ProDosimetry film digitizer (VIDAR Systems Corporation, Herndon, VA). A TomoScanner water tank (Standard Imaging) combined with a TomoElectrometer (Standard Imaging) was used to measure PDD curves and profiles.

#### D.1 Open fields for model commissioning

The commissioning of the VSM started with comparing the calculated and measured PDDs for each jaw size in the water tank. The high energy portion of the spectrum (Jeraj et al.'s spectrum^(16)^) was slightly modified to achieve the best match with PDD measurements. The open‐field PDD curves and profiles of the three jaw sizes (1.0, 2.5, and 5.0 cm) were used for commissioning. The voxel size of the transverse and longitudinal profiles, as well as PDDs, was set to $0.2\times 0.5\times 0.5\;{\text{cm}}^{3}$, $0.5\times 0.5\times 0.2\;{\text{cm}}^{3}$, and $0.5\times 0.2\times 0.5\;{\text{cm}}^{3}$, respectively, to achieve good resolution.

#### D.2 Accuracy in heterogeneous media

To evaluate the accuracy of the VSM in heterogeneous media, a previously studied geometric configuration was implemented.^32^, ^33^ The geometry of the test phantom consisted of two 5.0 cm thick slabs of water‐equivalent material, separated with one 15.0 cm thick slab of water‐composition material with density of 0.1 g/cm^3^. The jaw size was 2.5 cm and the central four leaves were open to form a $2.5\times 2.5\;{\text{cm}}^{2}$ field. The phantom was placed with the isocenter at 10 cm depth, which corresponded to a SSD of 75 cm. A static beam was used with zero degree gantry angle. To evaluate the accuracy of the VSM approach, we compared the results with those calculated by a well‐accepted MC package for tomotherapy, TomoPen,^17^, ^41^ which was based on PENELOPE^(22)^ and had been previously validated. As described earlier, TomoPen adopts the full MC model with precise mechanic drawings of a linac and is considered a gold standard in tomotherapy MC simulation.

#### D.3 MLC validation

The presented VSM MLC model was verified by simulating a Picket Fence pattern (T&G effect) and comparing with film measurements. A leaf sequence was created such that even leaves up to Leaf 34 were open and odd leaves starting from Leaf 35 were open, in order to test both even and odd leaves. The jaw size was 2.5 cm and the lateral field size was 40 cm. A zero degree static beam static couch calibration procedure was manually created in the treatment console station and the procedure was run for film measurements. EBT2 film was measured at the depth of 1.5 cm under solid water slabs with SSD of 85 cm. Robust film dosimetry was established in advance to convert film optical density to dose value.

#### D.4 Clinical case

Cylindrical Virtual Water phantom (also known as “Cheese Phantom”) (supplied by TomoTherapy Inc., Madison, WI) and film point dose measurements were employed for patient DQA verifications. A “Cheese Phantom” is a solid water cylinder with 30 cm in diameter and 18 cm in length. The prescanned CT image data of the phantom is 256×256×58 in dimension with a voxel size of $1.953\times 1.953\times 3\;{\text{mm}}^{3}$. A simple helical plan was created for determining MC dose calibration, with a cylinder shape PTV (2 cm in diameter and 6 cm in length) at the center of the phantom, with a prescription fraction dose of 2 Gy. The pitch was 0.22. The jaw size was 2.5 cm and the actual modulation factor was 1.785.

To demonstrate the capability of our VSM in verification and second checks for tomotherapy patient treatment plans, a complex head and neck (H&N) clinical case was studied, due its higher likelihood of having deviations between MC calculations and TPS CS dose algorithms in air cavities.^(27)^ The same voxel size and CT numbers to density table were implemented in the TPS. After simulation, the MC‐calculated dose distribution was imported into the TPS for generating DVH and film analysis. The study was carried out in the following two stages: first, a DQA plan on the cheese phantom was created, measured with EBT2 GAFCHROMIC films; second, dose distribution was compared for the patient treatment plan. The plan had two PTVs with a prescription of 60 Gy (PTV1) and 56 Gy (PTV2) in 30 fractions. The organs at risk (OARs) included spinal cord, brain stem, and esophagus. The MC simulated dose was compared with tomotherapy TPS‐calculated dose to demonstrate the impact of heterogeneity commonly seen in clinical cases.^17^, ^19^, ^20^, ^42^

## III. RESULTS

**Figure 5:**
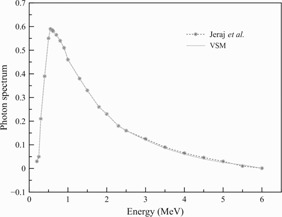
Photon energy spectrum used in the MC‐VSM simulations.

The code was executed with 10 computing nodes, simulating $5\times 10^{9}$ particles for each of the test runs. The statistical uncertainty was under 0.5% for each case in the study. Figure 5 shows the photon energy spectrum used in our MC simulations. Compared with Jeraj et al.'s spectrum,^(16)^ the spectrum used in our work was almost identical, except for the slightly modified spectral tail for the best match of the PDD and profile measurements.

**Figure 6:**
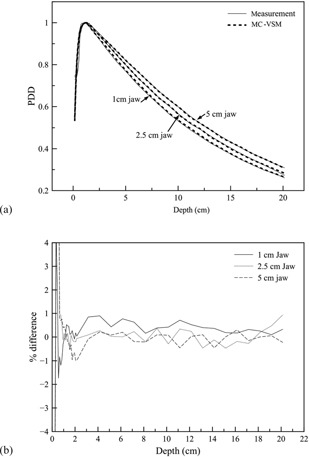
PDDs for the open fields with 40 cm×jaw sizes (1 cm, 2.5 cm, and 5 cm), respectively: (a) comparison between MC‐VSM and measurement; (b) percentage difference between MC‐VSM and measurement.

**Figure 7:**
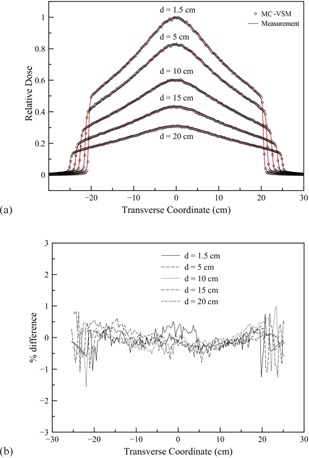
Transverse profiles for the open field $40\times 5\;{\text{cm}}^{2}$ at different depths: (a) comparison between MC‐VSM and measurement; (b) percentage difference between MC‐VSM and measurement.

**Figure 8:**
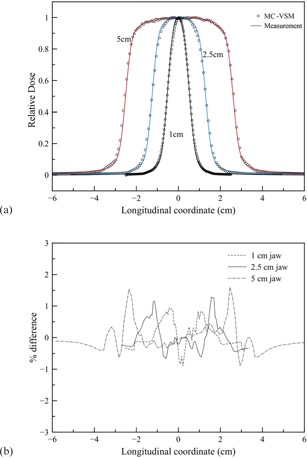
Longitudinal profiles for the three jaw sizes at depth of 1.5 cm in solid water: (a) comparison between MC‐VSM and measurement; (b) percentage difference between MC‐VSM and measurement.

**Figure 9:**
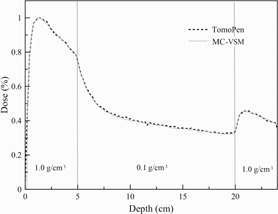
Comparison between MC‐VSM and TomoPen MC for the dose profile at the center axis of a heterogeneous media.

PDD comparisons and the corresponding percentage differences between measurements and MC‐VSM calculations for the open fields with 40 cm×jaw sizes (1 cm, 2.5 cm, and 5 cm) are shown in Figs. 6(a) and 6(b). Using 1%/1 mm gamma index (γ)^(43)^ acceptance criteria, 96%–98% points on the MC PDD curves agree with measurements. The discrepancies (up to 20%, not shown in the figure) are mostly at the buildup region (<5 mm), likely due to measurement inaccuracy resulted from the finite size of the cylindrical ion chamber in the buildup region.^(18)^ Figures 7(a) and 7(b) show transverse profile comparisons and the corresponding percentage differences between measurements and MC‐VSM calculations for the $40\times 5\;{\text{cm}}^{2}$ open field at various depths. The longitudinal profiles and percentage differences for the three jaw sizes at the depth of 1.5 cm are shown in Figs. 8(a) and 8(b), respectively. Good agreement (<1%) was achieved for both transverse and longitudinal profiles, except for the penumbra area (∼2%). For accuracy verification in heterogeneous media, Fig. 9 shows the comparison of dose distributions calculated with the MC‐VSM and the TomoPen MC package for the configuration described in the Materials & Methods section D.2 above. Overall, the MC‐VSM results agree with the TomoPen calculation within 1%.

**Figure 10:**
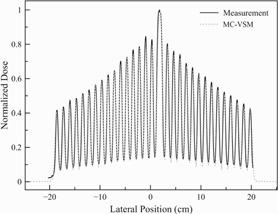
Comparison of the Picket Fence‐like MLC pattern between the MC‐VSM calculation and the film measurement for the 5 cm jaw.

For MLC validation, the comparison of film measurement and MC calculations is shown in Fig. 10. Using γ analysis^(43)^ with 2%/1 mm acceptance criteria, the passing rate was 96.4% comparing film measurements with MC‐VSM calculations (data spatial resolution was 0.3 mm and a total of 1142 points), which indicated accurate modeling of MLC leaves and the T&G effect.

**Figure 11:**
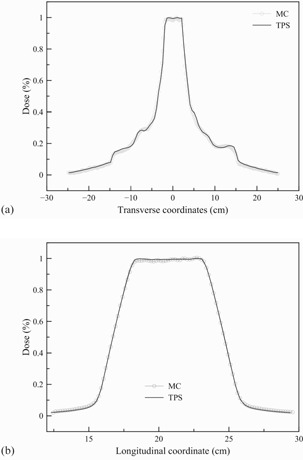
Comparison of the (a) transversal and (b) longitudinal dose profiles between the MC‐VSM calculation and the TPS for the calibration helical DQA plan.

For MC‐to‐dose calibration, a simple phantom plan was calculated in TPS to deliver a dose of 2 Gy to the center of the cheese phantom. Dose delivery was performed on the machine and confirmed by the A1SL ion chamber measurement. The calibration factor M was determined as $2.41\times 10^{11}$ (particles/s), which was used to convert MC calculation to radiation dose. Figures 11(a) and 11(b) show transverse and longitudinal dose profiles compared with the TPS results at the center of the PTV, respectively. The MC simulation agrees with the TPS result at a 99.5% gamma passing rate (γ [1%, 2 mm]) beyond the calibration point.

**Figure 12:**
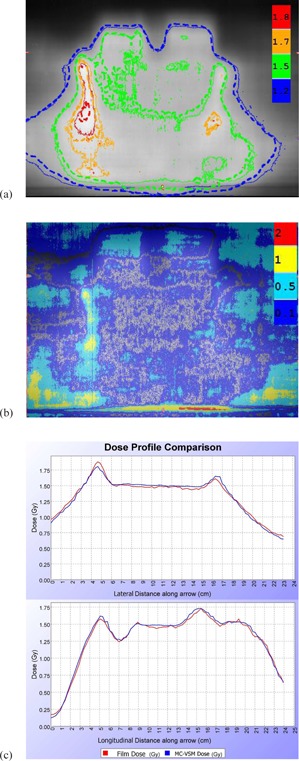
Comparison of the isodose lines (a) between MC‐VSM calculation result (dashed) and the film measurement (solid). Legend shows the isodose value (Gy). Gamma value map (b) calculated with 2%/2 mm criteria between MC‐VSM calculation and the film measurement. Legend shows the gamma value. Lateral and longitudinal profile comparison (c). Film measurement is shown in red and MC‐VSM calculation is shown in blue.

For the complex H&N case, PTV1 was prescribed a higher dose (60 Gy) with a total volume of 770 cc inside the PTV2 (56 Gy). In order to reduce the statistical uncertainty below 0.5%, $10^{11}$ particles were simulated on 10 computing nodes, resulting in a total simulation time of 1621.4 min. The MC‐VSM simulation for the DQA plan on a cheese phantom well agrees with EBT2 film measurements (Fig. 12(a)), with a high gamma passing rate using the 2%/2 mm criteria (except for the film edge), as shown in Fig. 12(b).

**Figure 13:**
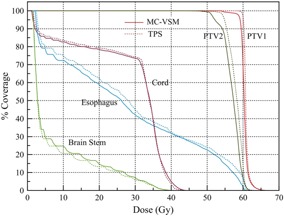
Comparison of dose obtained from MC‐VSM calculation and the TPS for the HN treatment plan. Isodose lines (a) (20 Gy, 30 Gy, 40 Gy, and 55 Gy) on an example slice. Thick lines represent the MC‐VSM and thin lines represent TPS. DVH curves (b) for the plan. Solid lines represent MC‐VSM and dotted lines represent TPS.

The isodose lines in heterogeneous HN patient plan in an example slice were illustrated in Fig. 13(a). The DVHs for PTV1, PTV2, spinal cord, brain stem, and esophagus for the HN patient plan were shown in Fig. 13(b). The values of mean dose comparing MC‐VSM vs. TPS were 60.4 Gy vs. 60.8 Gy for PTV1, 57.1 Gy vs. 58.0 Gy for PTV2, 7.96 Gy vs. 7.38 Gy for brain stem, 28.4 Gy vs. 28.7 Gy for spinal cord, and 27.8 Gy vs. 29.2 Gy for esophagus. The overall agreement between MC‐VSM and TPS for both PTVs and OARs was within 2% in average, with a maximum deviation for esophagus of 1.9 Gy (3% of the prescription dose) for $D_{50}$. However, a lower $D_{95}$ was observed for MC‐VSM (0.9 Gy or 1.7% of the prescribed dose for PTV1, 2.3 Gy or 4% of the prescribed dose for PTV2) than the TPS, which indicated that PTV coverage may be overestimated by TPS. This observation is consistent with literature.^3^, ^15^, ^27^, ^42^

## IV. DISCUSSION

Previous efforts of MC modeling of conventional linacs involve multiple virtual sources^30^, ^44^, ^45^, ^46^, ^47^ to simulate primary photons, scattered photons from the linac head and collimator, and contamination electrons. It has been demonstrated that the contribution of scattered photon for tomotherapy is much lower than conventional linac, due to its unique design.^(35)^ Therefore, our hypothesis is that the three‐source model can be simplified to a single‐source model. The present study confirmed that a single virtual source model with one primary photon component can be employed in MC to accurately calculate patient dose distributions. The study demonstrated the simplicity and effectiveness of the single‐source model for MC simulations of helical tomotherapy.

Tomotherapy adopts a “Gold Standard” beam model approach for machine commissioning. Each machine in the factory is adjusted through a process called “beam twinning”, to match the “Gold Standard” beam model and produce identical characteristic profiles such as PDDs, and transverse and longitudinal profiles. Therefore, the VSM we proposed can be applied to any “twinned” machine as long as the leaf filter is updated on‐site during machine commissioning, since the MLC leaf filter and leaf latency are machine‐specific and not included in the “Gold Standard” model. It is difficult to include them in the “Gold Standard” data since the MLC leaves produce unique fluence that is sensitive to the hardware and installation. More specifically, the leaf latency varies with the air pressure as the MLC is driven by compressed air.^(38)^

The MC MLC modeling in this work was approximated with the use of a leaf filter, which was employed in the CS‐based dose calculation for the tomotherapy TPS. The limitation of this approach is that the model cannot directly simulate the MLC leakage as seen in Zhao et al.^(18)^ Nevertheless, it accurately reproduces the MLC T&G effect. The MLC leakage is considered minimal (∼0.3%) compared to direct beams, thus not included in simulation for treatment planning.

This study presented an alternative approach for MC dose calculations for tomotherapy users who do not have access to the proprietary linac design specification. The “Gold Standard” beam model used by tomotherapy TPS is archived in the system and readily available to users. In addition, the VSM approach enables easy sharing of the beam model as the data storage requirement is small. We are currently in the process of releasing the VSM to the public domain.

## V. CONCLUSIONS

It has been shown that the VSM based on the TPS beam commissioning data can be accurately applied in the patient MC dose calculation for helical tomotherapy. An overall of <2% agreement can be achieved between MC calculations and measurements for static beam profiles and heterogeneous phantoms in the model commissioning procedures. An agreement of <3% was also obtained for clinical H&N plans. This novel approach does not require the detailed modeling of tomotherapy linac head, and it can be used in verification and second checks for patient treatment plans.

## ACKNOWLEDGMENTS

The author would like to thank Lydia Levinson Handsfield, M.S. for proofreading this paper. This work made use of the High Performance Computing Resource in the Core Facility for Advanced Research Computing at Case Western Reserve University.
